# Effect of a communication intervention on alcohol use during pregnancy in post conflict Northern Uganda: a quasi experimental study

**DOI:** 10.1186/s13011-022-00505-y

**Published:** 2022-12-12

**Authors:** Apophia Agiresaasi, Goretti Nassanga, Gakenia Wamuyu Maina, Juliet Kiguli, Elizabeth Nabiwemba, Noah Kiwanuka, Aggrey Mukose, Nazarius Mbona Tumwesigye

**Affiliations:** 1 Department of Epidemiology and Biostatistics School of Public Health, Makerere University College of Health Sciences, P.0 Box, 7062 Kampala, Uganda; 2 Department of Journalism and Communication, School of Language, Literature and Communication, Makerere University College of Social Sciences and Humanities, P.0 Box, 7062 Kampala, Uganda

**Keywords:** Alcohol use, Communication, Pregnancy, Antenatal care, Post conflict, Uganda

## Abstract

**Background:**

Alcohol use during pregnancy is a preventable risk factor for Fetal Alcohol Spectrum disorders. Psycho-social and educational interventions have been reported to enable women reduce alcohol intake levels during pregnancy and help improve some health outcomes of unhealthy alcohol use. We set out to assess the effect of a communication intervention on alcohol use during pregnancy in post conflict northern Uganda.

**Methods:**

The study employed a quasi - experimental design to assess the effect of a community health worker led communication strategy on pregnant women’s knowledge, attitudes and various patterns of alcohol use using Difference in Difference(DiD). 420 respondents were recruited at baseline as at endline.

**Results:**

The communication messages were significantly associated with reduced odds of binge drinking (*P* = 0.018; OR = 0.09; CI = 0.012–0.66). Also those who received the intervention were less likely to drink frequently (*P* = 0.80; OR = 0.75; 95%CI = 0.074–7.5) or be harmful alcohol users(*P* = 0.948). The intervention also positively influenced having fair (β =0.49;*P* = 0.217;RRR =1.63)or adequate knowledge(β = 0.89;*P* = 0.25;RRR = 2.44) and having positive(β = 0.37;RRR =1.44;*P* = 0.46) or fair attitude(β = 0.19;RRR = 1.21; *P* = 0.693) although not to a significant level.

**Conclusions:**

The communication intervention affected some patterns of alcohol use among pregnant women and not others. Our results contribute to existing evidence that communication interventions are a promising approach in reduction of alcohol exposed pregnancies. Interventions aimed at promoting alcohol abstinence during pregnancy should be implemented alongside other strategies that address factors that influence pregnant women to drink to achieve maximum results.

## Background

Prenatal alcohol use is an avoidable cause of birth defects and developmental disabilities termed Fetal Alcohol Spectrum Disorders (FASD) [1, 2]. There is no cure for FASD and it is irreversible but it can be completely prevented if women abstain from alcohol consumption while pregnant or when trying to conceive given that women often do not recognize they are pregnant until 4 to 6 weeks post conception [3, 4]. A safe level of alcohol exposure during pregnancy is not known for the fetus, and the evidence is inconclusive regarding the effects of low to moderate maternal alcohol consumption [5, 6]. Furthermore, data consistently show that although many women reduce their consumption or abstain from drinking alcohol during pregnancy, some do not [7, 8]. Thus many pregnancies remain alcohol exposed across the world.

Global estimates of alcohol use during pregnancy in the general population were recorded at 9.8% with large variability between countries [9]. The World Health Organization (WHO) surveys have classified Uganda among the leading alcohol consumers globally in the last two decades [10, 11]. Worse still, in Uganda maternal drinking during pregnancy in the general population was estimated at 20.5%, the highest in Africa [9]. In post conflict northern Uganda, a region scarred from a two decade civil war, 23.6% of women seeking Antenatal care services(ANC) reported alcohol use (any amount) during pregnancy while 29.3% of drinkers reported binge drinking [12]. The relatively high levels of drinking during pregnancy in the region have been attributed to poverty and the deeply engrained social - cultural norms that don’t only tolerate but also glamorize drinking [13].

Women who drink at high-risk levels during pregnancy have other contextual issues that need to be addressed and these include depression, abuse, homelessness and among others [14].Psychological and educational interventions have been reported to promote alcohol abstinence or reduction of alcohol intake during pregnancy [15]. More so, adequate epidemiologic evidence links reduced levels of alcohol consumption with a reduced risk for morbidity and mortality, providing indirect support that behavioral counseling/ educational interventions that reduce acute and sustained alcohol intake levels can help improve some health outcomes of unhealthy alcohol use [16].

Health and non - health professionals have an important role to play in the prevention of prenatal alcohol exposure. In California, community health workers were instrumental in implementing a brief intervention (10 to 15 minute) guided by a scripted manual in community settings with much success as women in the intervention group were 5 times more likely to report alcohol abstinence as compared to those in the control group [17]. More so women in the intervention group had higher birth weight, birth lengths and lower fetal mortality [17]. In Northern Cape South Africa, community health workers were credited for raising awareness on harms related to drinking during pregnancy and FASD levels were reportedly lower in post intervention areas [18]. But these studies are few and far between especially in Africa.

To bridge this gap, this study assessed the effect of a community health worker led communication strategy on knowledge, attitudes and the quantity and frequency of drinking during pregnancy in a post conflict environment.

## Methods

### Study area and design

The study employed a quasi - experimental study design. We used the design of non-equivalent groups [19–21].The study assessed the effect of a communication intervention that was implemented from February 2021 to March 2022. Prior to that, a baseline study had been conducted in the same area. Factors found to influence alcohol use during pregnancy during the baseline were useful in informing the intervention. Kitgum and Pader districts were selected as the control group and Gulu district as the intervention district. Gulu district was the epi-center of much of the fighting between the Lord’s Resistance Army LRA) and Uganda Peoples Defence Forces (UPDF) soldiers. Kitgum and Pader district also experienced socio economic disruptions and deaths resulting from the war. Agriculture is the main source of livelihood for people in the three districts.

### The communication intervention

The main communication goal was to increase knowledge, risk perception and improved practices related to alcohol consumption during pregnancy. The communication objectives were guided by Thomas W Valente [22] on the Hierarchy of Communication effects that postulates that when 80% of the population is exposed to the message, 60% will understand the message, 40% will approve the message, 20% will intend to Act and 5% will Act.

Specific objectives included 1) a 50% increase in number of women who know at least 4 dangers of drinking during pregnancy as compared to baseline; 2) At least 20% increase in number of women who believe alcohol use during pregnancy is important/very important in increasing risks to mother and baby during pregnancy and 3) By the end of the intervention, there will be a 5% increase in number of women aged 15–49 who have abandoned/reduced frequency and quantity of alcohol consumption compared to baseline findings.

Baseline study results [12, 13] were presented in a meeting during a community dialogue with men, women (target group) and health workers. In these meetings, a participatory approach was used in which stakeholders validated the study results and priorities were ranked with regard to which messages should be delivered. Later concepts were developed; communication materials were drafted with the Ministry of Health - Health Promotion Department. Messages were about chemicals contained in the alcohol, dangers of drinking during pregnancy, myths and misconceptions around alcohol use during pregnancy, factors that influence women to drink when pregnant and how they can stop it. Visual images to be included were also discussed with the stakeholders. Communication campaigns have been reported to be effective in raising awareness about drinking during pregnancy and that visual images can get the attention of the public towards understanding the consequences [18, 23]. These materials were translated to Acholi, the local dialect in the region by the Acholi language board.

Materials were then pretested using focus groups discussions with Village Health Team (VHTs) in the intervention district guided by a member from the district health department. The main points in the messages were summarized and VHTs made suggestions regarding the interpretation of messages, the font size, appropriateness of the images used, accuracy of the translation and the messaging. Revisions were made and a final leaflet made and printed.

Messages targeted women in reproductive age group as the primary audience but spouses, mother in-laws and other family members who directly influence the pregnant women’s decisions were also reached with messages. The sample size for the intervention was all women in the reproductive age group in Gulu district who are estimated to be 23% of the total population in the district according to UBOS 2018 projections. The VHTs visited all households with women of reproductive age group in their villages raising awareness about the dangers of drinking during pregnancy at least three times. These were supervised by health assistants/health inspectors.

We worked with 2 to 4 Community Health Workers known as Village Health Teams in Uganda (VHTs) per village depending on the size of the village and number of households therein. Each village has an average of about 30 to 80 households. Community health workers and other non -medical professionals have been instrumental in teaching women about dangers of maternal drinking. In De Aar and Upington, two areas heavily affected by FASD in the Northern Cape Province of South Africa, Community health workers taught women about FAS [18].

These VHTs were identified by health workers and trained by staff from the district health department. They disseminated these messages in their own villages where they hail from. The main mode of communication was interpersonal where VHTs moved from house to house raising awareness about the dangers of drinking during pregnancy. They also passed on these messages through already constituted groups such as village meetings, clan meetings, church gatherings and market days among others. This was deemed appropriate as some scholars have noted that communication interventions can target different levels of communities through wide varieties of mechanisms delivered by different modes of channels [24].

The VHTs were directly supervised by health assistants and filled in form showing number of households visited, numbers of women in those households, gatherings at which messages were disseminated, issues raised. These were signed and stamped by the village local council one chairman. These VHTs and the health assistants were paid a modest stipend agreed upon by the district health department and the research team each time they moved out to disseminate the messages.

### Monitoring the intervention

After the first phase of the intervention, monitoring was conducted by the first author and research assistants using a simple monitoring tool adopted from a social behaviour change communication manual [25]. During monitoring of the communication campaign, Gulu district(intervention area) was stratified into sub counties. Simple random sampling was used to select the sub-counties in which the monitoring was done using paper lots. At the second stage parishes within those sub-counties were randomly selected. The villages were listed on paper and selected using simple random sampling. At the third stage, in each of the strata, households were selected from the house hold lists provided by VHTS.

The required numbers of households were obtained randomly. The sampling unit was women of reproductive age group and other influential family members. The Lot Quality Assurance Sampling (LQAS) which is a low - cost random sampling approach to monitoring and evaluation was used. It has been used a lot to monitor health interventions [26]. We had a random sample of 19(It provides an acceptable level of error). 5 Sub-counties were randomly selected out of the 10 sub-counties in Gulu District. Out of these 15 parishes were be randomly selected. We had a total sample size of 285 respondents. We asked them if they had received any messages about drinking during pregnancy in the communities and what messages they recalled from that interaction. This mini survey informed the next phase of intervention. VHTs in the control group were facilitated once to visit households reminding pregnant women about antenatal care visits at health facilities.

### Sample size determination and sampling

With regard to the sample size for the endline we calculated the sample size by syntax; cluster sampsi, binomial detectable difference p1(.44) k(40) rho(.14) m [5].

Cluster-sampsi was used because treatment was assigned at cluster level yet outcome was measured at individual level.

**P1 =** Proportion in Population 1(control group).

**P2 =** Proportion in population 2(Intervention group).

**K =** Number of clusters in each arm.

**Rho** = Intra cluster correlation.

M = Sample size per cluster.

### Power calculations

We estimated that a sample of 420 women (210 from control and 210 from intervention) from the two per cluster arm was needed. A health facility was taken to represent a cluster. Five women were randomly selected per health facility. This implies a random selection of 42 health facilities from each arm that provided the adequate sample size of 420 women. This allowed us to detect 14–17 percentage improvement in women abandoning the consumption of alcohol during pregnancy. Power calculations were done using stata version 15 software that provided us 80% power at 95% significance.

We used multistage sampling whereby we stratified Acholi sub-region into 8 regions consisting of the 8 districts. We then used simple random sampling to select the three districts of Pader, Gulu and Kitgum from the 8 districts in Acholi sub-region using paper lots. At the second stage, the health facilities providing ANC services in the three districts were randomly selected. All categories of health facilities in a given stratum were catered for purposes of representativeness. Health facilities were grouped first according to district, then later, according to levels (hospital, Health centre IVs, Health centre IIIs and health centre IIs). After that, we obtained the sampling interval by dividing total number of health facilities in sampling frame divided by facilities to be included in the sample *n* = 84. To pick the first facility, we rounded off the sampling interval up to highest full number and selected any number from that. We then obtained a random number between 1 and 2 that was chosen using paper lots. To obtain the next facility we rounded up the sampling interval to the previous result. We continued with this process until all required number of facilities were selected.

At the third stage, in each of the strata, the required number of respondents by health facility category were obtained randomly from the available facilities of the category of interest using systematic random sampling with the starting point obtained by simple ballot. The sampling unit was women attending ANC services at health facilities in the three districts where we obtained 5 women within each cluster. Unlike at baseline where sampling was based on probability proportional to size at endline sampling was based on power calculations to ensure the sample was powered enough to derive the required outcomes from the study.

### Data collection

We interviewed women attending antenatal care at selected health facilities in Pader, Gulu and Kitgum districts in Post Conflict Northern Uganda. We assessed respondents for eligibility to participate in the interview. To be eligible, women had to be in the reproductive age group (at least 15 year and above) and be at least be (8 to 36 weeks) gestation and Ability to speak Luo and or English and be willing to participate. Women who refused to participate and women who are drank/very sick or required emergency care were not interviewed. Having sought ANC services, selected respondents were interviewed. We collected data using a computerized, Internet-based survey tool (ODK) using tablets. Women were asked about alcohol use (any amount and type). Quantity and frequency of alcohol consumed was also recorded. We also used the World Health Organization’ Alcohol Use Disorders Identification Test (AUDIT) questionnaire to assess alcohol related problems [27].

Amount consumed was quantified in terms of standard drinks WHO WHS(World Health Survey) defines a standard drink as one containing at least 8 to 13 g of pure alcohol [28]. The following measures were taken as one standard drink. i) a 285-ml bottle or can of beer, (ii) a 120-ml glass of wine (factory distilled or locally brewed), and (iii) a 30-ml glass/tot of a spirit or gin (factory distilled or locally brewed). For local brews a standard drink was measured using Alcohol by Volume for beverages where ABV was known [28]. The questionnaires were pretested in Amuru district. The questions in the AUDIT questionnaire were modified to reflect alcohol consumption during pregnancy.

We interviewed women about their Socio demographic, socio behavioural and other characteristics. In addition, we asked women about their knowledge of dangers regarding drinking during pregnancy and perceptions about the same. Women who reported 4 or more dangers of drinking during pregnancy were categorised as having adequate knowledge. Those who mentioned 2 to 3 effects were categorised as having fair knowledge and those who were able to mention 0–1 were regarded as having poor knowledge.

#### Data management and analysis

Data management and analysis was carried out using SPSS software program for windows version 21 SPSS Inc. Chicago USA as well as stata 15. Data entry errors were minimized by having a customized check program inbuilt in the data entry program and constant supervision by the researchers. Data was also checked for completeness and accuracy before leaving the facility and as such no data were missing. Frequency tables were generated for key variables in line with the analysis plan. Other forms of descriptive analysis included testing for association (e.g using chi-square test or t-test where applicable) between outcome variables as proxy measures of “alcohol related behaviour” and testing for significance change between intervention (Gulu district) and control (Kitgum and Pader). Such proxy measures of “behaviour change” included but were not limited to: knowledge, attitudes, frequency and quantity of alcohol consumed.

Difference-in-Difference (DiD) was used to measure the impact from the proxy measures of “reduced alcohol consumption”. For instance, where frequency of alcohol use was used to measure behaviour change then we computed the baseline value for both the intervention (Gulu) and control area (Pader and Kitgum) among the respondents. For instance let M_1_ and M_0_ represent frequency of alcohol use score for (Gulu) and (Pader and Kitgum) at baseline respectively while H_1_ and H_0_ are scores at endline. Then the DiD computation [29] was subjected to 5% level of significance test is represented as:

1 $$\textrm{DiD}=\left({\textrm{H}}_{1-}{\textrm{M}}_1\right)-\left({\textrm{H}}_{0-}{\textrm{M}}_0\right)$$

DiD = Difference in Difference.

H_1 =_ Observation for intervention group at endline.

M_1 =_ Observation for intervention group at baseline.

H_0_ = Observation for Control group at endline.

M_0 =_ Observation for control group at baseline.

### Multivariable analysis (DiD approach)

The DiD was preferred because it was not possible to randomize the intervention area and control area. We estimated the intention-to-treat (ITT) effect of the intervention, which captures the impact of being part of each group as opposed to actually receiving treatment. The ITT provides the average impact of the intervention on the population in general targeted by the program. The regression model (Eq. 2) controlled for key socio-economic and demographic characteristics e.g. sex, age, residence of respondents. The main statistical tests used included binary logistic, multinomial and OLS regression. Binary logistic regression was used in determining the effect of the intervention on alcohol dependence(non-alcoholic dependant or alcoholic dependent), alcohol use any amount (no or yes) frequent drinking(drinking at least three times a week i.e. frequent and infrequent), Binge drinking (light or Binge drinker).

Ordinary Least Squares Regression (OLS) was used to estimate effect of the intervention on harmful and hazardous drinking (The dependant variable was continuous/numeric).

The Multinomial Logistic Regression was used in determining the effect of the intervention on knowledge (poor, fair and adequate) and attitudes(poor, fair and positive)

2 $${Y}_{it v}={\sigma}_0+\kern0.5em {Treat}_{it v}{\sigma}_1+\kern0.5em {Post}_t{\sigma}_2+{ Treat XPost}_{it v}{\sigma}_3+\kern0.5em {X}_{it}\beta +\kern0.5em {\upsilon}_v+{u}_{it v};$$

Regarding the Audit, the hazardous alcohol use (questions 1 to 3) cut-off point was put at 6. The dependence (questions 4 to 6) cut-off was 4 and the harmful use (questions 7 to 10) cutoff was 7 points. Scores ≥8 were considered an indicator of problematic alcohol use [30].

Study participants were categorized as abstainers if they did not report alcohol consumption since on-set of pregnancy. Forms of alcohol consumption were 1) alcohol use (any amount); 2) Frequent drinking(at least three times a week); 3) Binge drinking (Consumption of four drinks at one sitting).

## Results

### Characteristics of study participants

Overall, 420 eligible women attending ANC in selected health facilities in Gulu, Kitgum and Pader districts in Northern Uganda were interviewed at end line as at baseline. Socio demographic characteristics of respondents at endline are described herein.

About a third of the respondents 132(31.4%) belonged to the 21 to 25 age-group. Most respondents 244(58.1%) reported Primary education as their highest level of academic attainment. The vast majority 374(89.05%) were either married or cohabiting and with regard to place of residence a significant proportion 255(60.7%) of the respondents hailed from rural areas. Slightly more than half 237(56.4%) belonged to the catholic religion and peasant farmers dominated the sample 114(27.1%) followed by house wives *n* = 109(25.9%). This is presented in Table 1.

**Table 1:** Socio Demographic Characteristics of Respondents at Baseline and End-Line

			Group category				*p*-value
			Control		Intervention		
	Baseline	15–20	54	29.0%	56	23.9%	
		21–25	57	30.6%	69	29.5%	
Age		26–30	41	22.0%	64	27.4%	0.416
		31–35	22	11.8%	35	15.0%	
		36–45	12	6.5%	10	4.3%	
	Endline	15–20	57	27.1%	56	26.7%	
		21–25	71	33.8%	61	29.0%	
		26–30	48	22.9%	52	24.8%	0.091
		31–35	18	8.6%	33	15.7%	
		36–45	16	7.6%	8	3.8%	
	Baseline	No formal education	20	10.8%	23	9.8%	
		Primary	118	63.4%	119	50.9%	0.022
Highest Level of Education Attained		Secondary	32	17.2%	69	29.5%	
		Higher	16	8.6%	23	9.8%	
	Endline	No formal education	22	10.5%	16	7.6%	
		Primary	123	58.6%	121	57.6%	0.625
		Secondary	49	23.3%	58	27.6%	
		Higher	16	7.6%	15	7.1%	
	Baseline	Married	56	30.1%	101	43.2%	
		Single/separated/divorced	11	5.9%	17	7.3%	< 0.001
Marital status		Cohabiting	119	64.0%	94	40.2%	
		Other	0	0.0%	22	9.4%	
	Endline	Married	63	30.0%	105	50.0%	
		Single/separated/divorced	28	13.3%	18	8.6%	< 0.001
		Cohabiting	119	56.7%	87	41.4%	
		Other	0	0.0%	0	0.0%	
	Baseline	Rural	140	75.3%	103	44.0%	
		urban	43	23.1%	113	48.3%	< 0.001
Residence		Peri urban	3	1.6%	18	7.7%	
	Endline	Rural	143	68.1%	112	53.3%	
		urban	29	13.8%	72	34.3%	< 0.001
		Peri urban	38	18.1%	26	12.4%	
	Baseline	Catholic	111	59.7%	141	60.3%	
		Protestant	47	25.3%	58	24.8%	
		Muslim	7	3.8%	7	3.0%	0.971
Religion		Pentecostal	21	11.3%	28	12.0%	
		Others	0	0.0%	0	0.0%	
Occupation	Endline	Catholic	116	55.2%	121	57.6%	
		Protestant	61	29.0%	42	20.0%	
		Muslim	6	2.9%	3	1.4%	0.043
		Pentecostal	27	12.9%	42	20.0%	
		Others	0	0.0%	2	1.0%	
	Baseline	Civil Servant	0	0.0%	0	0.0%	
		Farmer	104	55.9%	82	35.0%	
		Housewife	28	15.1%	57	24.4%	
		Retail business	21	11.3%	48	20.5%	` 0.001
		Cross border trader	0	0.0%	0	0.0%	
		Fish folk	0	0.0%	0	0.0%	
		Other(specify)	33	17.7%	47	20.1%	
	Endline	Civil Servant	6	2.9%	7	3.3%	
		Farmer	72	34.3%	42	20.0%	
		Housewife	49	23.3%	60	28.6%	
		Retail business	35	16.7%	55	26.2%	0.016
		Cross border trader	0	0.0%	0	0.0%	
		Fish folk	1	.5%	2	1.0%	
		Other(specify)	47	22.4%	44	21.0%	

Descriptive statistics were used to obtain numbers and percentages of respondents and their socio demographic characteristics. Slightly more than half of the respondents were catholic. Two thirds resided in rural areas. About a half had attained up to primary level education and about a third were aged between 21 to 25

Other socio - behavioural and obstetric characteristics of respondents were captured; many of the women reported one sexual partner. Very few women reported to have ever smoked or used tobacco(1.0%) and less than 1% reported to have ever used marijuana. Most respondents at end-line were pregnant for the first time in both the control 73(34.8%) and intervention 68(32.4%) and majority were in their second trimester as at baseline. This is presented in Table 2.

**Table 2:** Social Behavioral and Obstetric Characteristics of Respondents at Baseline and End-Line

			Group category				*p*-value
			Control		Intervention		
	Baseline	Non gravida	55	29.6%	42	17.9%	
		One	27	14.5%	57	24.4%	
		Two	34	18.3%	59	25.2%	0.001
		Three	44	23.7%	40	17.1%	
		Four	12	6.5%	26	11.1%	
Live children		Five+	14	7.5%	10	4.3%	
	Endline	Non gravida	73	34.8%	68	32.4%	
		One	46	21.9%	41	19.5%	
		Two	36	17.1%	58	27.6%	0.150
		Three	21	10.0%	20	9.5%	
		Four	21	10.0%	16	7.6%	
		Five+	13	6.2%	7	3.3%	
	Baseline	First trimester	48	25.8%	62	26.5%	
		Second trimester	101	54.3%	124	53.0%	0.965
Trimester		Third trimester	37	19.9%	48	20.5%	
	Endline	First trimester	35	16.7%	68	32.4%	
		Second trimester	95	45.2%	91	43.3%	< 0.001
		Third trimester	80	38.1%	51	24.3%	
	Baseline	One	172	92.5%	207	88.5%	0.069
Sex Partners in last twelve months		Two or more	14	7.5%	27	11.5%	
	Endline	One	184	87.6%	184	87.6%	1.000
		Two or more	26	12.4%	26	12.4%	
	Baseline	Yes	0	0.0%	3	1.3%	0.121
Ever Smoked or used Tobacco		No	186	100.0%	231	98.7%	
	Endline	Yes	2	1.0%	1	.5%	0.562
		No	208	99.0%	209	99.5%	
	Baseline	Yes	1	.5%	1	.4%	0.870
Ever Used Marijuana or other Types of Drugs		No	185	99.5%	233	99.6%	
	Endline	Yes	0	0.0%	1	.5%	0.317
		No	210	100.0%	209	99.5%	

Descriptive statistics were used to obtain numbers and percentages of respondents and their socio behavioural and obstetric characteristics. About a third of respondents were non-gravida. Most were in their second trimester of pregnancy and the vast majority reported one sex partner in last twelve months preceding the survey

### Association between the intervention and drinking patterns, knowledge and attitudes

Participants were asked at end line and at baseline about various patterns of alcohol use during the current pregnancy. Cross tabulation results reveal that the intervention was associated with reduction in alcohol consumption. The most notable impact was recorded with regard to binge drinking (consumption of four or more drinks at one sitting), where binging significantly (*P* = 0.04) reduced by 38.3 percentage points. Communication messages were also significantly (*P* < 0.001) associated with reduced consumption of alcohol (any amount) by 6.22 percentage points. However, there was a non-significant (*P* = 0.17) increase in frequent drinking by 6.55 percentage points but this was not attributed to the intervention. These are presented in Table 3.

**Table 3:** Impact of Intervention on Drinking Patterns (Cross Tabulation Results)

				Group category			Size of the impact (%)	*p*-value
				Contro	Intervention			
	Baseline	No	80	43.0%	114	48.7%		0.244
Ever Consumed Alcohol		Yes	106	57.0%	120	51.3%		
	Endline	No	84	40.0%	110	52.4%		0.011
		Yes	126	60.0%	100	47.6%		
Currently Drinking Alcohol	Baseline	No	47	44.3%	81	67.5%		< 0.001
		Yes	59	55.7%	39	32.5%	−6.22%	
	Endline	No	60	47.6%	77	77.0%		< 0.001
		Yes	66	52.4%	23	23.0%		
		Currently consuming	59	31.7%	40	17.1%		
	Baseline	Light drinkers	43	72.9%	19	47.5%		0.010
Binge drinking		Binge drinkers	16	27.1%	21	52.5%		
	Endline	Light drinkers	46	69.7%	19	82.6%	−38.30%	0.043
		Binge drinkers	20	30.3%	4	17.4%		
Frequency of alcohol consumption	Baseline	Infrequent	33	55.9%	30	75.0%		0.053
		Frequent	26	44.1%	10	25.0%		
	Endline	Infrequent	52	78.8%	21	91.3%	6.55%	0.178
		Frequent	14	21.2%	2	8.7%		
**AUDIT C SCORES**								
				Group category			size of the impact (%)	*p*-value
				Control	Intervention			
Hazardous drinkers	Baseline	Non hazardous	101	95.3%	117	97.5%		
	Endline	Hazardous drinker	5	4.7%	3	2.5%	0.46%	0.368
Hazardous drinkers	Baseline	Non hazardous	120	95.2%	97	97.0%		0.501
	Endline	Hazardous drinker	6	4.8%	3	3.0%		
Alcohol dependence	Baseline	Non alcoholic dependent	57	96.6%	39	95.1%		
	Endline	Alcohol dependence	2	3.4%	2	4.9%	−1.30%	0.709
Alcohol dependence	Baseline	Non alcoholic dependent	54	91.5%	21	91.3%		0.974
	Endline	Alcohol dependence	5	8.5%	2	8.7%		
Harmful use	Baseline	Harmless use	59	100.0%	41	100.0%		
Harmful use	Endline	Harmless use	59	100.0%	23	100.0%		

The Chi-square test was used to establish the effect of the intervention on various drinking patterns. The intervention was associated with reduced alcohol use(any amount), alcohol dependence and binge drinking

Regarding the AUDIT C scores, cross tabulation results show a decline in number of respondents reporting alcohol dependence by an insignificant (*P* = 0.97) proportion of 1.3 percentage point difference and only a 0.46 percentage point increase in participants insignificantly (*P* = 0.50)reporting hazardous drinking albeit these were not associated with the communication messages. These are presented in Table 3.

Respondents were asked a number of questions to gauge their knowledge concerning maternal alcohol use during pregnancy; Following the intervention, there was an 11.98 percentage point rise in women with adequate knowledge (women who could mention four or more danger of drinking during pregnancy) and this increase was highly (*P* = 0.001) significant. This is presented in Table 4.

**Table 4:** Impact of the Intervention on Respondents Knowledge and Attitudes(Crosstabulations)

			Group Category					*P*-Value
			Control		Intervention		Size of Impact	
	Baseline	Poor knowledge	108	58.1%	126	53.8%		
		Fair knowledge	73	39.2%	103	44.0%		0.599
Knowledge of the dangers of alcohol use during pregnancy		Adequate	**5**	2.7%	5	2.1%		
	Endline	Poor knowledge	112	53.3%	75	35.7%	11.98%	0.001
		Fair knowledge	51	24.3%	64	30.5%		
		Adequate	**47**	22.4%	71	33.8%		
Has no effect on the baby	Baseline	Agree	19	10.2%	30	12.8%		
		Disagree	124	66.7%	177	75.6%		0.006
		Don’t know	43	23.1%	27	11.5%	−6.90%	
	Endline	Agree	25	11.9%	16	7.6%		
		Disagree	150	71.4%	172	81.9%		0.040
		Don’t know	35	16.7%	22	10.5%		
Should be stopped completely	Baseline	Agree	140	75.3%	194	82.9%		
		Disagree	21	11.3%	30	12.8%	7.70%	0.003
		Don’t know	25	13.4%	10	4.3%		
	Endline	Agree	144	68.6%	172	81.9%		
		Disagree	17	8.1%	13	6.2%		0.005
		Don’t know	49	23.3%	25	11.9%		
Can be good for pregnancy	Baseline	Agree	3	1.6%	21	9.0%		
		Disagree	135	72.6%	192	82.1%	−14.98%	< 0.001
		Don’t know	48	25.8%	21	9.0%		
	Endline	Agree	27	12.9%	11	5.2%		
		Disagree	129	61.4%	170	81.0%		< 0.001
		Don’t know	54	25.7%	29	13.8%		
Can be used occasionally during pregnancy	Baseline	Agree	21	11.3%	31	13.2%		
		Disagree	137	73.7%	190	81.2%	−12.43%	0.005
		Don’t know	28	15.1%	13	5.6%		
	Endline	Agree	41	19.5%	19	9.0%		
		Disagree	113	53.8%	147	70.0%		0.001
		Don’t know	56	26.7%	44	21.0%		
Can be used after attaining a certain number of months of pregnancy	Baseline	Agree	20	10.8%	30	12.8%		
		Disagree	134	72.0%	188	80.3%		0.004
		Don’t know	32	17.2%	16	6.8%	−10.64%	
	Endline	Agree	36	17.1%	18	8.6%		
		Disagree	100	47.6%	135	64.3%		0.001
		Don’t know	74	35.2%	57	27.1%		
Do you agree that pregnant women shdnt drink	Baseline	Agree	144	77.4%	190	81.2%		
		Disagree	13	7.0%	25	10.7%	8.60%	0.033
		Dont know	29	15.6%	19	8.1%		
	Endline	Agree	143	68.1%	169	80.5%		
		Disagree	21	10.0%	14	6.7%		0.014
		Dont know	46	21.9%	27	12.9%		
Importance of alcohol in increasing chances of health risks for wmn and babies	Baseline	Poor attitude	73	39.2%	61	26.1%		
		Fair	27	14.5%	31	13.2%		0.008
		Positive	86	46.2%	142	60.7%	7.46%	
	Endline	Poor attitude	88	41.9%	49	23.3%		
		Fair	43	20.5%	36	17.1%		< 0.001
		Positive	79	37.6%	125	59.5%		

The chi-square test was used to estimate the association between the communication intervention and respondent’s knowledge and attitudes towards drinking during pregnancy. It was significantly associated with increased levels of adequate knowledge and other proxy measure of knowledge and increased positive attitude

A number of other proxy measures of knowledge appear to have improved and were significantly associated with the communication messages. These include; a 8.60 percentage point increase in number of women in agreement that pregnant women should not drink at all(*P* = 0.014); a 12.43 percentage point decline in women saying alcohol can be used occasionally during pregnancy(P = 0.001); a 10.64 percentage point decline in women mentioning that alcohol use is okay after attaining a certain number of months of pregnancy(P = 0.001); a 6.90 percentage point decline in women who noted that alcohol has no effect on the baby *P* = 0.040) and a 14.98 percentage point decline in women stating that alcohol can be good for pregnancy(*P* < 0.001). This is presented in Table 4.

With regard to attitudes, there was a7.46 percentage point increase in number of women with positive attitudes (felt alcohol was important/very important increasing health risks to the mother and baby during pregnancy) and this was significantly (P < 0.001) attributed to the communication intervention. This is presented in Table 4.

### Effect of the intervention on drinking patterns, knowledge and attitudes

Binary logistic regression results reveal that women who received the intervention had reduced odds of indulging in binge drinking (*P* = 0.015; OR = 0.16; CI =0.038–0.707). More so, with the introduction of the intervention, alcohol use (any amount) and frequent drinking (drinking 3 times a week) decreased (*P* = 0.395; OR = 0.71; CI = 0.32–1.57) and (*P* = 0.85; OR = 0.84; CI = 0.14–5.04) respectively although not to a significant level. These are presented in table 5.

**Table 5:** Effect of Intervention on Drinking Patterns (Alcohol Use (Any Amount), Binge Drinking and Frequent Drinking)Binary Logistic Regression

Alcohol use(any amount)					
Currently Drinking	Coef	Odds Ratio	P > z	[95% Conf.	Interval]
Time					
Endline	−0.13	0.88	0.618	0.52	1.47
Group					
Intervention	−0.96	0.38	0.001	0.22	0.66
time#group					
endline#intervention	−0.35	0.71	0.395	0.32	1.57
_cons	0.23	1.26	0.245	0.86	1.84
**Frequent Drinking**					
Time					
Endline	−1.07	0.34	0.007	0.16	0.75
Group					
Intervention	−0.86	0.42	0.056	0.18	1.02
time#group					
endline#intervention	−0.18	0.84	0.845	0.14	5.04
_cons	−0.24	0.79	0.363	0.47	1.32
**Binge drinking**					
Time		1.17	0.695	0.54	2.54
Endline	0.16				
Group					
Intervention	1.09	2.97	0.012	1.28	6.92
time#group					
endline#intervention	−1.81	0.16	0.015	0.04	0.71
_cons	0.99	0.37	0.001	0.21	0.66

The binary logistic regression was used to estimate the effect of the intervention on binge, frequent and alcohol use(any amount). The intervention was only significantly associated with reduced odds of binge drinking after putting into account time and belonging of either the intervention or control group

Concerning the AUDIT scores, communication messages reduced chances of being a dependant drinker but not to a significant extent (*P* = 0.794; OR = 0.704;95% CI: 0.05–9.82). In the results of OLS regression, there was an AVERAGE decline in being a harmful alcohol user depicted by the negative coefficient of − 0.181 and being a hazardous alcohol user (β = − 0.172) although not to a significant extent (*P* > 0.05). This is presented in Table 6 and 7.

**Table 6:** Effect of Intervention on Audit Scores (Alcohol Dependence, Hazardous Use And Harmful Use) Binary Logistic Regression And OLS Regression

Alcohol Dependence					
Alcohol Dependence	Coef	Odds Ratio	P > z	[95% Conf.	Interval]
Time					
Endline	0.97	2.6	0.258	0.49	14.18
Group					
Intervention	0.379	1.5	0.710	0.20	10.82
time#group					
endline#intervention	− 0.351	0.7	0.794	0.05	9.83
_cons	−3.35	0.0	0.000	0.01	0.14
**Harmful Alcohol Use**					
Time					
Endline	0.186		0.379	−0.23	0.60
Group					
Intervention	0.748		0.002	0.29	1.21
time#group					
endline#intervention	−0.181		0.621	−0.90	0.54
_cons	0.203		0.175	− 0.09	0.50
**Hazardous Alcohol Use**					
Time			0.821	−0.50	0.40
Endline	−0.052				
Group			0.019	−0.99	−0.09
Intervention	−0.541				
time#group			0.599	−0.81	0.47
endline#intervention	−0.172		0.000	1.09	1.75
_cons	1.425				

The Binary logistic regression was used to estimate the effect of the intervention on Alcohol dependence while the OLS regression was used to estimate effect of intervention on harmful and harzadous alcohol use. The intervention was not significantly associated with being an alcohol dependant, harmful or harzadous alcohol user

**Table 7:** Effect of Intervention on Respondents Knowledge and Attitudes (Multi-Nomial Logistic Regression

Knowledge						
	Fair			Adequate		
	Coef.	RRR	P > z	Coef.	RRR	P > z
time (Ref = Baseline)						
Endline	−0.395	0.674	0.082	2.204	9.064	< 0.001
group (Ref = Control)						
Intervention	0.190	1.209	0.345	−0.154	0.857	0.811
time*group						
endline#intervention	0.438	1.550	0.162	0.968	2.632	0.160
_cons	−0.392	0.676	0.010	−3.073	0.046	< 0.001
Number of obs = 840						
LR chi2(6) = 140.18						
Prob > chi2 = 0.0000						
Pseudo R2 = 0.0834						
**Attitudes**						
	**Fair attitude**			**Positive attitude**		
time (Ref = Baseline)						
Endline	0.2784859	1.321	0.340	−0.272	0.762	0.221
group (Ref = Control)						
Intervention	0.3177359	1.374	0.314	0.681	1.976	0.002
time*group						
endline#intervention	0.0900994	1.094	0.833	0.363	1.438	0.253
_cons	−0.9946226	0.370	0.000	0.164	1.178	0.303
Number of obs = 840						
LR chi2(6) = 140.18						
Prob > chi2 = 0.0000						
Pseudo R2 = 0.0834						

The multinomial logistic regression was used to estimate effect of the intervention on knowledge and attitude towards drinking during pregnancy and it had no significant influence in differentiating those with adequate knowledge vs poor knowledge or those with positive attitude vs poor attitude

With regard to knowledge, a multinomial logistic regression was employed to predict the effect of the intervention on the probability of falling in any of the three knowledge categories; Poor knowledge, fair knowledge and adequate knowledge. The intervention had a positive influence in increasing knowledge from poor to either fair (β = 0.438, RRR = 1.55, P > 0.05) or Adequate knowledge (β = 0.968, RRR = 2.632, P > 0.05). However, the intervention had no significant influence in differentiating women with Fair or adequate knowledge relative to their counterpart with poor knowledge. This is presented in Table 8.

**Table 8:** Effect of the Intervention on Various Patterns of drinking(Multivariate Analysis)

Drinking Patterns	Alcohol use (Any amount)	Binge drinking	Frequent drinking	Alcohol Dependence	Hazardous drinking	Harmful alcohol use
**Variable**	**OR [95% CI]**	**OR [95% CI]**	**OR(95% CI)**	**OR [95% CI]**	**OR [95% CI]**	**Coeff[95% CI]**
**Age Group**						
21–25	1.26(0.53 to 2.99)	0.76(0.12 to 4.48	0.49(0.06to 3.93)	0.30(0.01 to 9.82)	2.58(0.14 to 45.20)	−0.20(− 0.88 to 0.4)
26–30	1.37(0.50 to 3.73)	2.03(0.33 to 12.13	0.86 (0.11 to 6.74)	0.84(0.04 to 14.99)	2.40(0.12 to 46.83)	− 0.38(− 1.13 to 0.37)
31–35	1.73(0.55 to 5.46)	6.41(0.86 to 47.40	1.2(0.13 to 11.36)	0.76(0.03 to 17.45)	7.04(0.3 to 156.21)	−0.29(−1.15 to 0.57)
36–45	1.02(0.24 to 4.33)	1.73(0.19to 15.72)	0.4(0.04 to 4.43)		7.49(0.25 to 217.99)	−0.19(−1.16 to 0.78)
**Live Children**						
One	1.16(0.45 to 2.97)	9.8(1.30 to 73.89	64(3.06to 133.81)	3.67(0.12 to106.66)	1.29(0.07 to 24.32)	0.17(−0.58 to 0.92)
Two	1.20(0.46 to 3.14	3.5(0.40 to 32.07)	32.8(1.40 to77.42)	2.32(0.10 to 52.10)	2.03(0.10 to 39.97)	−0.12(− 0.98 to 0.73)
Three	1.09(0.36 to 3.32)	3.2(0.33 to 30.66)	34.07(1.48 to 78.73)		0.70(0.03 to 18.98)	−0.16(−1.07 to 0.76)
Four	1.47(0.44 to 4.91	4.2(0.48 to 37.13)	45.4(1.94 to 1062.5)	0.76(0.03 to 16.97)	1.43(0.05 to 38.31)	−0.03(− 0.92 to 0.86)
Five+	1.55(0.36 to 6.63)	2.06(0.20to 21.22)	26.6(1.07 to 658.47)		1.02(0.03 to 35.89)	0.002(−0.99 to 0.99)
**Residence**						
Urban	0.75(0.41 to 1.39)	0.99(0.33 to 2.94	0.93(0.28 to 3.094)	0.88(0.08 to 9.44)	1.20 (0.31 to 4.59)	0.27(− 0.23 to 0.77)
Peri urban	0.58(0.24 to 1.43)	1.63(0.38 to 6.88	0.64(0.11 to 3.53)	1.56(0.18 to 13.29)	1.09 (0.16 to 7.05)	0.24(−0.39 to 0.87)
**Sex Partners in last twelve months**						
Two or more	5.17(2.12 to 12.63)	1.59(0.58 to 4.34)	4.6(1.55 to 14.03)	0.44(0.05 to 3.68)	5.95(1.26 to 28.11)	0.33(−0.12 to 0.77)
**Knowledge of anything that could harm baby during pregnancy**						
Yes	1.32(0.65 to 2.66)	0.22(0.07 to 0.68)	1.09(0.32 to 3.76)	2.85(0.40 to 20.16)	3.17 (0.49 to 20.25)	−0.10(− 0.57 to 0.36)
**Attitude**						
Fair attitude	0.15(0.07 to 0.34)	4.8(1.16 to 19.90)	0.60(0.09 to 3.74)	0.68(0.04 to 10.63)	1.88 (0.30 to 11.70)	0.04(−0.62 to 0.70)
Positive attitude	0.46(0.20 to 1.036)	6.63(1.64to 26.77)	3.2(0.72 to 14.94)	0.46(0.03 to 8.05)	1.45 (0.20 to 10.36)	0.13(−0.49 to 0.77)
**My family would not approve of me drinking during pregnancy**						
Agree	0.06(0.03 to 0.16)	0.42 (0.17 to 1.01)	0.24(0.08 to 0.65)	0.11(0.01 to 0.84)	0.08 (0.03 to 0.32)	−0.19(− 0.59 to 0.21)
**Ever received information on alcohol use during pregnancy**						
Yes	0.49(0.26to 0.92)	0.56(0.19 to 1.61)	0.77(0.22 to 2.62)	0.25(0.02 to 2.71)	0.83 (0.20 to 3.49)	0.22(−0.26 to 0.70)
**Knowledge**						
Fair knowledge	0.67(0.34 to 1.35)	1.40(0.42 to 4.61)	9.54(2.28 to 39.90)	3.65(0.28 to 47.71)	3.33 (0.69 to 15.97)	0.58(0.01 to 1.15)
Adequate	0.68(0.24 to 1.84)	5.62(1.01to 31.25)	3.54(0.48 to 25.80)		3.05 (0.36 to 25.35	−0.14(−1.005 to 0.71)
Time						
Endline	0.74(0.37 to 1.51)	0.79(0.27 to 2.29)	0.15(0.04 to 0.54)	1.26(0.11 to 13.40)	0.46(0.09 to 2.24)	0.22(−0.26 to 0.72)
Group						
Intervention	0.23(0.10 to 0.50)	3.02(0.84to 10.79)	0.21(0.05 to 0.90)	0.27(0.01 to 6.24)	0.24 (0.04 to 1.42)	0.48(−0.10 to 1.073)
time#group						
Endline#intervention	1.48(0.53 to 4.10)	0.09(0.01to 0.66)*	0.75(0.07 to 7.54)	7.08(0.08 to 578.14)	2.61 (0.24 to 27.68)	− 0.03(− 0.85 to 0.79)
Cons	24.6(7.47 to 81.52)	0.12(0.02 to 0.61)	(0.03(0.003to 0.44)	0.25(0.01 to 10.49)	0.01 (0.001 to 0.21)	0.31(−0.37 to 0.99)

A multivariable logistic regression was used to estimate effect of the intervention on various patterns of drinking. The communication intervention was only significant with regard to binge drinking

We also used a multinomial logistic regression to predict the effect of the intervention on the probability of falling in any of the three attitude categories; Negative attitude, fair attitude and positive attitude. Results reveal that the intervention had a positive influence of enhancing attitudes from poor to fair (β = 0.090, RRR = 1.09, *P* = 0.833) or positive (β =0.363,RRR = 1.44,*P* = 0.25) but it had no significant influence in differentiating women with fair or positive attitude relative to their counterparts with poor attitude. This is presented in Table 6 and 7.

### Multivariable results

After controlling for a host of socio-economic and demographic factors, the intervention remained significantly associated with reduced chances of binge drinking (*P* = 0.018; OR = 0.09; CI = 0.012–0.66). Also those who received the intervention were less likely to drink frequently although this was not significantly linked to the intervention (*P* = 0.80 OR = 0.75; 95%CI = 0.074–7.5).Surprisingly, the intervention was not linked to reduced odds of alcohol use(any amount)(*P* = 0.447; OR = 1.48;95%CI =0.53–4.10). This is presented in Table 8.

Concerning the AUDIT scores, results show that respondents who received the communication messages had reduced odds of being harmful alcohol users (*P* = 0.948). Receiving the communication messages was not associated with alcohol dependence (*P* = 0.384; OR = 7.08; 95%CI = 0.086–578.1 and hazardous drinking (*P* = 0.425; OR = 2.61;95%CI = 0.24–27.68). This is presented in Table 8.

After controlling for a host of other factors, multinomial logistic regression results reveal that the intervention positively influenced having fair (β =0.49;*P* = 0.217;RRR =1.63;)or adequate knowledge(Could mention at least 4 dangers of drinking during pregnancy) (β = 0.89;*P* = 0.25;RRR = 2.44) However it was not significant in differentiating women with fair or adequate knowledge relative to those poor knowledge. This is presented in Table 9.

**Table 9:** Effect of the Intervention on Knowledge (Poor Knowledge is the Base Outcome)

	Fair knowledge				Adequate Knowledge			
	RRR	P > |z|	[95%	Conf. Interval]	RRR	P > |z|	[95%	Conf. Interval]
**Age group**								
21–25	1.45	0.203	−0.2	0.945	1.96	0.173	−0.295	1.644
26–30	1.32	0.431	−0.407	0.955	1.68	0.384	− 0.646	1.678
31–35	2.06	0.09	− 0.113	1.556	3.03	0.11	−0.252	2.469
36–45	1.05	0.939	−1.128	1.221	0.74	0.765	−2.227	1.637
**Living Children**								
One	0.67	0.186	−1.008	0.196	0.57	0.28	−1.6	0.463
Two	0.72	0.337	−0.99	0.339	0.98	0.975	−1.086	1.053
Three	0.67	0.32	−1.172	0.383	0.78	0.708	−1.508	1.023
Four	0.3	0.012	−2.168	−0.271	0.54	0.425	−2.129	0.898
five+	0.3	0.057	−2.462	0.038	0.55	0.564	−2.642	1.441
**Residence**								
Urban	1.05	0.832	−0.389	0.484	0.79	0.519	−0.956	0.483
Peri urban	1.56	0.217	−0.261	1.152	0.75	0.564	−1.271	0.693
**Number of Sex partners in the last twelve months**								
Two or more	1.03	0.925	−0.633	0.697	0.26	0.046	−2.696	−0.023
**Knowledge of anything that could harm baby during pregnancy**								
Yes	2.07	0.006	0.21	1.247	20.19	0	1.415	4.595
**Attitude**								
Fair attitude	2.03	0.018	0.122	1.291	2.63	0.441	−1.489	3.42
Positive attitude	15.5	0	2.177	3.305	551.14	0	4.238	8.386
**My family members wouldn’t approve of me drinking during pregnancy**								
Agree	0.93	0.839	−0.779	0.632	0.58	0.429	−1.916	0.814
**Ever received information on alcohol use during pregnancy**								
Yes	1.72	0.028	0.058	1.023	0.7	0.345	−1.086	0.379
**Time**								
Endline	1.3	0.4	−0.347	0.869	30.03	0	2.24	4.564
**Group**								
Intervention	0.7	0.193	−0.887	0.179	0.5	0.318	−2.045	0.665
**Time#Group**								
Endline#intervention	1.63	0.217	−0.288	1.268	2.44	0.256	−0.649	2.433
_cons	0.07	0	−3.487	−1.712	0	0	−12.903	−6.884
Number of obs = 840								
LR chi2(40) = 665.51								
Prob> chi2 = 0.0000								
Pseudo R2 = 0.3961								

Multinomial logistic regression was used to estimate the effect of the intervention on knowledge. It was not significant in differentiating women with fair or adequate knowledge relative to those poor knowledge

Similarly, results show that having received the messages on dangers of drinking during pregnancy was positively linked to having fair(felt alcohol was moderately important increasing health risks to the mother and baby during pregnancy) (β = 0.19;RRR = 1.21; *P* = 0.693) or positive attitude(felt alcohol was important/very important increasing health risks to the mother and baby during pregnancy)(β = 0.37;RRR =1.44;*P* = 0.46). None the less, the intervention was not significant in differentiating respondents with fair or positive attitude relative to those with poor attitude. This is presented in Table 10.

**Table 10:** Effect of Intervention on Attitudes (Negative Attitude is the Base Outcome)

		Fairattitude			Positiveattitude			
	RRR	P > |z|	[95%	Conf. Interval]	RRR	P > |z|	[95%	Conf. Interval]
**Knowledge**								
fair knowledge	2.19	0.009	0.198	1.374	15.74	0.000	2.177	3.335
Adequate	3.92	0.278	−1.102	3.835	832.95	0.000	4.601	8.848
**Age Group**								
21–25	1.76	0.119	−0.146	1.276	0.95	0.886	−0.793	0.685
26–30	2.30	0.054	−0.016	1.683	0.85	0.717	−1.051	0.723
31–35	1.71	0.320	−0.520	1.593	0.51	0.234	−1.764	0.431
36–45	2.97	0.134	−0.335	2.514	1.55	0.548	−0.991	1.866
**Living Children**								
One	0.60	0.182	−1.256	0.239	0.47	0.062	−1.544	0.039
Two	0.51	0.113	−1.514	0.160	0.42	0.050	−1.750	0.001
Three	0.64	0.394	−1.456	0.574	1.06	0.912	−0.995	1.115
Four	0.50	0.233	−1.821	0.443	1.12	0.856	−1.073	1.292
five+	0.20	0.040	−3.158	−0.073	0.55	0.414	−2.062	0.849
**Residence**								
Urban	1.45	0.205	−0.202	0.944	1.37	0.292	−0.271	0.900
Peri urban	0.77	0.575	−1.166	0.648	1.89	0.146	−0.223	1.501
**Number of Sex partners in last twelve months**								
Two or more	0.48	0.071	−1.512	0.062	0.52	0.097	−1.420	0.117
**Knowledge of anything that could harm baby during pregnancy**								
Yes	2.04	0.012	0.158	1.265	2.95	0.000	0.494	1.671
**My family wouldn’t approve of me drinking during pregnancy**								
Agree	2.20	0.024	0.104	1.473	16.10	0.000	1.844	3.714
**Ever received information on drinking during pregnancy**								
Yes	3.21	0.000	0.636	1.694	6.06	0.000	1.247	2.357
**Time**								
Endline	0.90	0.751	−0.769	0.554	0.19	0.000	−2.411	−0.928
**Group**								
Intervention	1.22	0.584	−0.516	0.915	2.12	0.034	0.055	1.446
**Time#group**								
Endline#intervention	1.21	0.693	−0.752	1.131	1.44	0.465	−0.616	1.348
_cons	0.09	0.000	−3.381	−1.530	0.01	0.000	−5.558	−3.217
Number of obs = 840								
LR chi2(40) = 681.42								
Prob > chi2 = 0.0000								

A Multinomial logistic regression was used to estimate the effect of the intervention on Attitude. It was not significant in differentiating women with fair or positive attitude relative to those with poor attitude

## Discussion

Results of this study show that the communication intervention on the dangers of alcohol use during pregnancy was significantly associated with reduced odds of binge drinking, a drinking pattern linked to Alcohol-exposed pregnancies (AEP), the direct cause of fetal alcohol spectrum disorders (FASD) [31–33]. This is in agreement with previous studies conducted in the region. A Community health worker led communication strategy in the Northern Cape Province of South Africa resulted in reduction in FASD rates and significant increase in knowledge on harmful effects of alcohol [18].

More so, in this interventional study on effects of a communication intervention on drinking during pregnancy, we observed that women exposed to the intervention were less likely to drink frequently and had reduced odds of being hazardous or harmful drinkers although not to a significant level. This is not dissimilar to previous studies employing psychosocial/ education interventions to reduce alcohol harms during pregnancy. Related studies have noted that promotion of alcohol abstinence alone had limited potential to impact behavior and should be done alongside other strategies meant to avert unwanted/unplanned pregnancies [34]. The other explanation for the non significant differences in hazardous, harmful and frequent drinking between the intervention and control arms may be attributed to the small numbers of women reporting these patterns of alcohol use.

It was also surprising that VHT home visits did not affect alcohol use (any amount)demonstrated by non-significant statistical tests between the control and intervention arms especially after putting into account a host of other factors. A similar study conducted in Southern California registered significant decline in alcohol use among those who received a 10–15 minute brief intervention administered by non - medical professionals as compared to those in the control group [17]. A qualitative inquiry might be helpful in explaining this phenomenon.

After controlling for other factors, the communication intervention positively impacted knowledge and attitudes towards maternal alcohol use during pregnancy but not to a significant extent.

This study is important because it is one of the few studies that have attempted to reduce alcohol consumption among pregnant women in Sub Saharan Africa and increase our understanding of alcohol use interventions in a post conflict setting. These results need to be interpreted in the context of the following limitations. We used self-reported measures of alcohol consumption that may be prone to recall and social desirability response biases. However, we tried to mitigate this by comparing with data from other sources or previous related studies. The best way to validate the self-report would be to use alcohol biomarkers.

Also, the study used the Difference in Difference (DiD) method to measure impact of the interventions and thus may not have easily estimated the role played by other prevailing factors that may affect differences in trends. Nonetheless, a multivariable linear regression was used to try and take into account a host of other factors. Both volume and concentration of alcohol consumed may be especially difficult to estimate where homebrews are common introducing bias. We used WHO World Health Service tool to define a standard alcoholic drink as one that contains as containing between 8 and 13 g of ethanol for commercial beverages whose alcohol content is known. The women interviewed at the baseline were different from the ones interviewed at endline as the intervention was evaluated after 12 months but all women in the reproductive age group in the intervention district were exposed to the communication messages and they bore similar characteristics as the women in the baseline. We did a simple random sample from a larger population and not a series of nested samples, a sampling design that could have created nonzero intraclass correlations within strata. As a result, the data may not be strictly independent.

## Conclusion

The aim of this study was to evaluate the effect of a community health worker led communication intervention on alcohol use during pregnancy. Results of this study confirm that we can reduce the volume of alcohol consumed by women through raising awareness on its dangers. The communication messages on the dangers of alcohol during pregnancy affected some forms of alcohol use among pregnant women and not others. The intervention accomplished to some extent the communication objectives such as 6.22 percentage point decline in women drinking alcohol (any amount) and the 38.3 percentage point decline in women reporting binge drinking against the 5% set target. With regard to attitudes, the intervention wasn’t able to realise the set target of 20% as there was only a 7.46 percentage point increase in number of women with positive attitudes (felt alcohol was important/very important increasing health risks to the mother and baby during pregnancy).Also, the intervention fell short of realising the 60% target of women understanding the message as there was only a 11.98 percentage point rise in women with adequate knowledge. Our results contribute to existing evidence that communication interventions are a promising approach in reduction of alcohol exposed pregnancies. Interventions aimed at promoting alcohol abstinence during pregnancy should be implemented alongside other strategies that address other factors that influence women to drink alcohol to achieve maximum results.

## Data Availability

All relevant data are within the manuscript. If further data needed, it could be accessed from the corresponding author upon request via email at agiresaasi@gmail.com.

## Abbreviations

ANC: Antenatal Care
AUDIT: Alcohol Use Disorders Identification Test
DHO: District Health Officer
FASD: Fetal Alcohol Spectrum Disorders
LBW: Low Birth Weight
LRA: Lord’s Resistance Army
MoH: Ministry of Health
ODK: Open Data Kit
OR: Odds Ratio
RRR: Relative Risk Ratio
SPSS: Statistical Package for Social Sciences
UNCST: Uganda National Council for Science and Technology
WHO: World Health Organization
